# Prevalence and associated risk factors for childhood strabismus in Lhasa, Tibet, China: a cross-sectional, school-based study

**DOI:** 10.1186/s12886-020-01732-2

**Published:** 2020-11-25

**Authors:** Hailong He, Jing Fu, Zhaojun Meng, Weiwei Chen, Lei Li, Xinyu Zhao

**Affiliations:** 1 Beijing Tongren Eye Center, Beijing Tongren Hospital, Capital Medical University; Beijing Ophthalmology & Visual Sciences Key Laboratory, No.1, Dong Jiao Min Xiang Street, Dongcheng District, Beijing, 100730 China; 2 Beijing Institute of Ophthalmology, Beijing, China; 3 Department of Epidemiology and Biostatistics, Institute of Basic Medical Sciences Chinese Academy of Medical Sciences, School of Basic Medicine Peking Union Medical College, Beijing, China

**Keywords:** Strabismus, Prevalence, Children, Epidemiology

## Abstract

**Background:**

To estimate the prevalence of strabismus and associated risk factors among grade one school children in Lhasa, Tibet, China.

**Methods:**

The Lhasa Childhood Eye Study (LCES) was a cross-sectional, school-based childhood study conducted in Grade one students from primary schools in Lhasa, Tibet, China. Comprehensive ophthalmic examinations and basic systemic examinations were evaluated. A questionnaire survey containing information about children, as well as parents’ information, was sent to the corresponding parents of eligible children. The prevalence of strabismus and its 95% confidence interval was estimated. Univariate and multivariate logistic regression analyses were conducted to determine the associated risk factors.

**Results:**

A total of 1942 eligible grade one students were enrolled, of which 1856 participants completed all examinations. The average age was 6.83 ± 0.46 years, 53% of participants were boys and 1762 were the Tibetan Minority. Over all, the prevalence of strabismus was 68/1856 (3.7%) (95%CI: 2.81,4.52), with no difference between the ages, genders, ethnicities and body mass index, while tilting one’s head when writing may be a risk factor for strabismus (*P* = 0.004). Strabismus students had mean best corrected visual acuity of 0.16 ± 0.28(LogMAR), over 50% patients with esotropia were hyperopic, and participants who had stereopsis impairments showed a significant difference between esotropia and exotropia (*P =* 0.026).

**Conclusions:**

The prevalence of strabismus in LCES was 3.7%, which is higher than previous reports from Chinese childhood epidemiology studies. Strabismus is a common contributing factor to amblyopia. Tilting one’s head when writing may be a risk factor. Esotropia is more likely to affect stereopsis and be associated with the refractive state of hyperopia.

**Trial registration:**

The study has finished the clinical registration on Chinese Clinical Trial Registry. (http://www.chictr.org.cn, ChiCTR1900026693).

**Supplementary Information:**

The online version contains supplementary material available at 10.1186/s12886-020-01732-2.

## Background

Strabismus, also called squint, is a condition of misalignment of the visual axes of the eyes, and was first known as eye misalignment since the Hippocratic era [1]. It can adversely affect not only binocular single vision, like simultaneous perception, fusion and stereopsis, but also cosmetic impairment, which can cause significant psychosocial consequences [2, 3] Strabismus is a common contributing factor to amblyopia in children, which will persist into adulthood if not treated in time [4]. Hence, early detection and treatment should be considered for strabismus and amblyopia, especially in preschool children, to maximize binocular potential and to improve outcomes [5, 6].

An accurate epidemiology of disease will help in evaluating the screening results, improving effective treatment strategies and guiding the allocation of medical resources [7]. However, although the prevalence of strabismus in school-based studies have been estimated in recent years, the prevalence of various studies differs. Screening for eye diseases in school-aged children has largely been done in large cities, with very few information available in remote areas, like Tibet at a high-altitude plateau area. Impaired vision in early childhood can have a profound impact on a child’s development. Screening in childhood has become an important part of the children’s eye health program and should be done early [8, 9].

Tibet is a region in Asia covering much of the Tibetan Plateau, with an average elevation of 5000 m. The atmospheric conditions are hypobaric, with thin air, high numbers of sunshine hours and strong ultraviolet radiation [10]. To our knowledge, although some epidemiological surveys of common eye diseases have been carried out in recent years, they lacked longitudinal investigation on eye diseases of school-age children in the Tibetan plateau areas [11]. The purpose of this survey was to assess the prevalence and progressive nature of strabismus in native Tibetan children, to determine its associated risk factors and to evaluate the demand for eye care service in Lhasa Prefecture, Tibet Autonomous Region, China.

## Methods

### Study design

The Lhasa Childhood Eye Study (LCES) was a cross-sectional, school-based, longitudinal cohort study conducted in Lhasa, Tibet, China. The study was mainly designed to estimate the prevalence and associated risk factors of ocular diseases in school-age children during a 5-year follow-up (Chinese Clinical Trial Registry [ChiCTR], Identifier: ChiCTR1900026693). Ethics committee approval was obtained from the Institutional Review Board of Beijing Tongren Hospital, Capital Medical University (TRECKY2019–146) in accordance with the Declaration of Helsinki principles. The first visit of LCES was between September 2019 to October 2019. Written informed consent forms were obtained and signed by all parents or guardians before the examinations. The procedures were modified from the Anyang childhood eye Study (ACES) conducted in central China [12].

### Participants

There are in total 28 elementary schools in Lhasa. Officials from the health and education departments of Lhasa summoned the principals of the 28 schools before recruitment, to inform them the LCES would be conducted from 2019 to 2024. Twenty-seven out of the 28 elementary schools in Lhasa were available to join and were stratified into three levels based on the evaluation of local government. Voluntary Grade one students who had been living in Lhasa city for at least half a year and would continue to live there for at least 5 years until they enter different middle schools were included in LCES. Individuals suffering from mental illness or other medical conditions who were unable to cooperate with the baseline survey were excluded. According to geographical characteristics and required sample size, 1943 Grade one students of seven primary schools were randomly sampled by stratified cluster sampling. All the clusters were numbered according to their locations and were randomly selected using a random numbers table. Participants were followed for 5 years until they entered different middle schools and could withdraw from the study at anytime for any reason.

### Parental questionnaire

The questionnaires were mainly derived from the version of that used in the ACES [12] and were modified to make sure the questionnaires were culturally appropriate and linguistically accurate. The questionnaire contained information about indoor and outdoor activity, history of the birth and ophthalmic treatment, and habits of reading, writing, living, eating and so on, as well as parents’ information such as refractive and socioeconomic status, education, pregnancy history, medical records and other aspects. For example, the questionnaire would ask 1)‘What is your child’s current preferred writing posture?’ and 2)‘As far as you remember, did your child prefer to tilt his head when he/she was writing?’ with the following options: Yes or NO or Not sure.

### Procedures

#### Systemic examinations

Blood pressure and heart rate were measured using a digital automatic blood pressure monitor (HK-808, HSH, Shenzhen, China). Oxygen saturation was measured using a digital fingertip pulse oximeter (YX301, YUWELL, Jiangsu, China). Height and weight (without shoes or heavy clothing) were determined using a weighing scale. Body Mass Index (BMI, BMI = weight/height^2^ kg/m^2^) was also calculated.

#### Ophthalmic examinations

The comprehensive eye examination was performed by a team of two optometrists and four ophthalmologists from Beijing Tongren Hospital who were trained and certified using standardized study protocols.

#### Distant and near visual acuity

Uncorrected and presenting distant visual acuity (VA) were measured for the right eye and left eye using Lea Symbols ETDRS 3 m set charts (250,300, Goodlite, IL, USA) at a distance of 3 m based on the instructions and standard procedure. The last line attempted, combined with the number of mistakes made on that and previous lines, was used to calculate a letter-by-letter logMAR VA score. Pinhole and best-corrected distant VA (BCVA) were obtained after subjective refraction test for students with uncorrected distance VA<20/20 (>LogMAR 0.0).

#### Refraction

Objective refraction was measured before and after cycloplegia using an autorefractor (KR-800, Topcon, Tokyo, Japan). The cycloplegic procedure for each student was to first administer one drop of topical anesthetic agent (Alcaine, Alcon), followed by two drops of 1% cyclopentolate (Alcon) and one drop of Mydrin P (Santen, Japan) given 5 min separately. Thirty minutes after the last drop was instilled, a third drop of cyclopentolate was administered if the pupillary light reflex was still present or the pupil size was less than 6.0 mm.

#### Ocular movements

Nine directions of gaze including primary, secondary and tertiary were examined and recorded by asking students to fix on a moving penlight without moving their heads.

#### Ocular dominance

Students were asked to hold a card with a central hole and fixate on a distant object while holding their head stationary. The examiner covered the students’ eyes one after the other and repeated it three times to determine the dominant eye.

#### Cover test

Unilateral and alternating cover tests were performed at far (6 m) and near (33 cm) to detect heterotropia and heterophoria by an experienced pediatric ophthalmologist under natural conditions (with and without spectacles), respectively, and the magnitude of the deviation was measured using loose prisms. The prism was placed with the base along the reversed direction of deviation and was adjusted until no movement could be detected.

#### Stereopsis

Stereo Fly Test (S0001, STEREO, USA) was used to quantitatively measure stereo acuity for students at 40 cm.

### Diseases definition and classification

Strabismus was defined if any heterotropia was present at near or distance, with or without spectacles and then classified according to the primary direction of the tropia [13].

Depending upon the clinic-etiological features, primary exotropia was classified into basic non-specific type (exotropia approximately equal for near and distance), convergence insufficiency type (exotropia greater for near than distance, ≥10 prism diopters [PD]), and divergence excess (exotropia greater for distance than near, ≥10 PD). Esotropia was classified into accommodative esotropia (due to overaction of convergence associated with accommodation reflex) and non-accommodative esotropia (all those primary esodeviations in which the amount of deviation is not affected by the state of accommodation and roughly equal in amount at distance and near fixation) [14].

Micro-strabismus was defined as a deviation of<10 PD in the presence of demonstrable binocular vision on the Lang II test. In the absence of demonstrable binocular vision, a deviation of this magnitude was classified simply as strabismus [15].

### Statistical analysis

The prevalence of strabismus was calculated and analyzed as the percentile of the number of individuals with different types of strabismus to the total number of individuals evaluated. Continuous variables were given as the mean ± standard deviation (SD), and categorical variables were given as the absolute value and relative frequency. Prevalence of strabismus and its 95% confidence interval (CI) was estimated using a general linear model. Risk factors associated with high level strabismus were evaluated using a linear regression model. The potential risk factors including general characters like age, gender, ethnicity, BMI, heart rate, oxygen saturation, and habits of reading, living, eating, as well as parents’ information such as pregnancy history and so on. The analyses were implemented with SAS software version 9.4 (SAS institute Inc. Cary, NC). χ2 tests and *t*-tests were also used. A *p*-value of < 0.05 was considered statistically significant.

## Results

### General information

A total of 1942 Grade one students were enrolled, of which 1902 (97.94%) were eligible for LCES according to the inclusion and exclusion criteria. A subset of 1856 of the remaining eligible individuals completed all examinations, giving a response rate of 97.58%. For the entire group of analyzable participants, 1762 (94.93%) students were the Tibetan Minority, 85 (4.58%) were Han Majority and nine (0.49%) were other minority nationalities. The average age was 6.83 ± 0.46 years and 984 (53.02%) of the participants were boys (Table 1). The mean BCVA measured by LogMAR was 0.05 ± 0.11 and 0.05 ± 0.10 for right and left eye, respectively. Cycloplegic spherical equivalent (SE) was + 1.07 ± 0.92 diopters (D). 1192 (64.22%) of the participants’ ocular dominance eyes were right eyes, 664 were left eye dominant, and none of them showed no dominance preference.

**Table 1:** Characteristics of participants of LCES (*N* = 1856)

Items	Values
Age, years (mean ± sd)	6.83 ± 0.46
Gender, *n*(%)	
Boy	984 (53%)
Girl	872 (47%)
Ethnic categories, *n*(%)	
Tibetan Minority	1762 (94.9%)
Han Majority	85 (4.6%)
Others Majorities	9 (0.5%)
Height, cm, (mean ± sd)	120.55 ± 5.52
Weight, kg, (mean ± sd)	22.96 ± 3.69
BMI, kg/m^2^, (mean ± sd)	15.74 ± 1.80
Heart rate, beat, (mean ± sd)	95.27 ± 14.27
Oxygen saturation, %, (mean ± sd)	92.68 ± 3.07
BCVA, LogMAR, (mean ± sd)	
Right eyes	0.05 ± 0.11
Left eyes	0.05 ± 0.10
Cycloplegic SE, D, (mean ± sd)	+ 1.07 ± 0.92
Ocular dominance eyes, *n*(%)	
Right eyes	1192 (64.2%)
Left eyes	664 (35.8%)

### Prevalence of strabismus

**Fig. 1:**
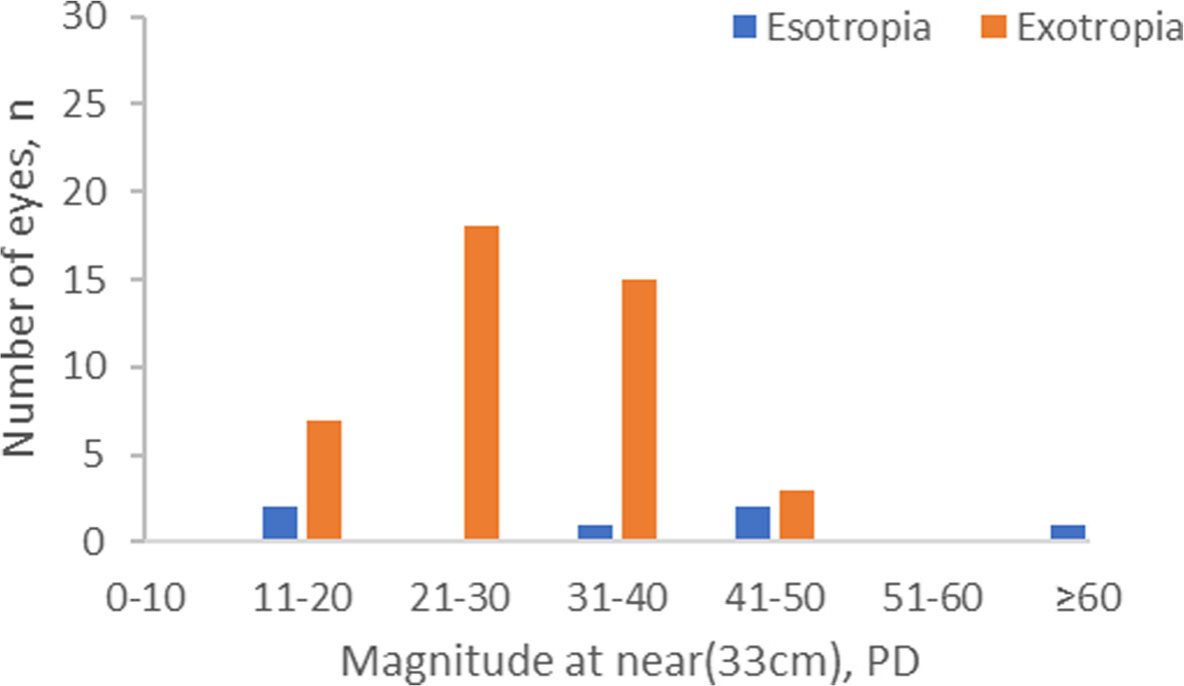
Distribution of the magnitude of exotropia and esotropia at 33 cm

**Fig. 2:**
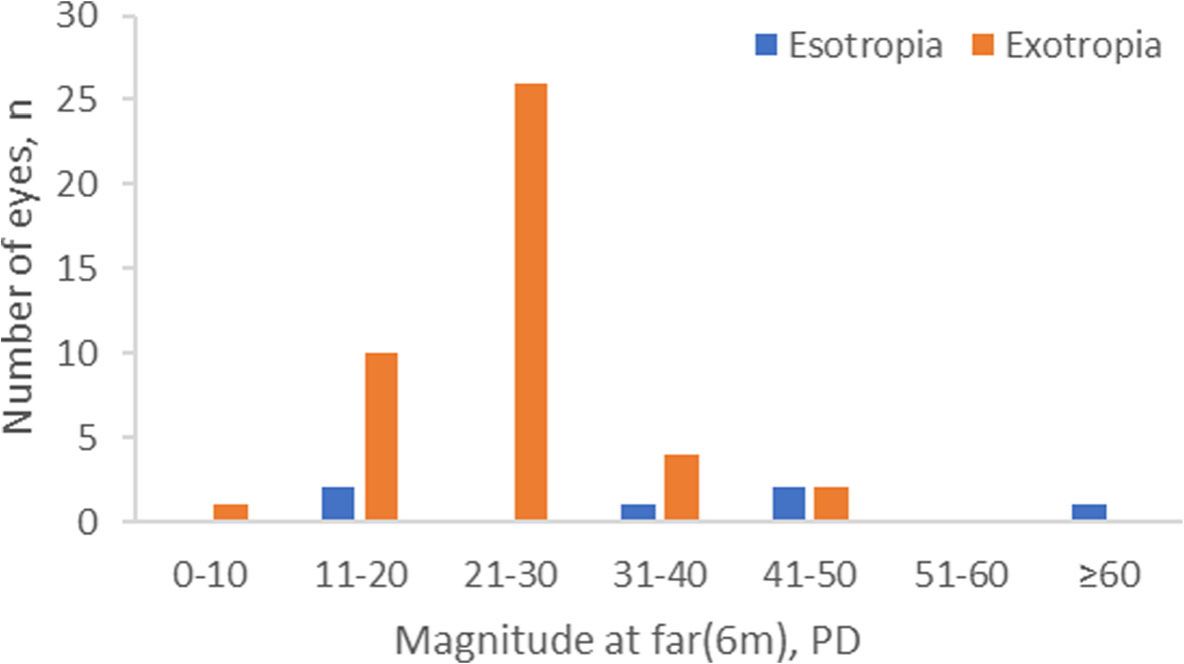
Distribution of the magnitude of exotropia and esotropia at 6 m

Based on the comprehensive eye examinations of the 1856 Grade one students, the overall prevalence of strabismus was 3.66% (95%CI, 2.81–4.52%). Of the 68 students with strabismus, the most frequent form of strabismus was exotropia (*n* = 43, 2.32%), followed by eight (0.43%) with esotropia, with an exotropia: esotropia ratio of 5.38:1, two (0.11%) with microtropia (one esotropia and one exotropia), eight (0.43%) with vertical deviation combining horizontal strabismus, seven (0.38%) with exotropia combining superior oblique muscle paralysis (SOP), and one (0.05%) with exotropia combining double depressor paralysis (DDP). Three cases (0.16%) had pure vertical strabismus, and all of these had SOP. Four (0.22%) special strabismus cases were diagnosed. Two had Duane’s retraction syndrome, one had dissociated vertical deviation (DVD) combining oblique muscle dysfunction and one had nystagmus due to congenital cataract (Table 2). The magnitude of exotropia and esotropia is shown in Fig. 1 and Fig. 2. Eleven students (16.18%) with strabismus also had amblyopia; eight (11.76%) were esotropia, two were exotropia and one was vertical deviation combining horizontal strabismus.

**Table 2:** Prevalence of strabismus and subtypes (*N* = 1856)

Classification	*n* (%)
Exotropia	43 (2.3)
Basic non-specific type	31 (1.7)
Divergence excess	1 (0.1)
Convergence insufficiency type	11 (0.6)
Esotropia	8 (0.4)
Accommodative esotropia	5 (0.3)
Non-accommodative esotropias	1 (0.1)
Others	2 (0.1)
Vertical deviation combining horizontal strabismus	8 (0.4)
Exotropia combining SOP	7 (0.4)
Exotropia combining DDP	1 (0.1)
Vertical strabismus	3 (0.2)
SOP	3 (0.2)
Microtropia	2 (0.1)
Others	4 (0.2)
Duane syndrome	2 (0.1)
DVD combining oblique muscle dysfunction	1 (0.1)
Nystagmus for congenital cataract	1 (0.1)
Total	68 (3.7%)

There was no significant difference of BCVA between the two eyes (*P* = 0. 59) for the entire group. The mean BCVA of the strabismus (0.16 ± 028) and non-strabismus (0.04 ± 0.09) children had significant difference *(P = 0.0008)*. Among 1192 participants’ with right-eye dominance, there were 40 with strabismus, while in the 664 left-eye dominant eyes, there were 28 with strabismus. The effect of ocular dominance between strabismus and non-strabismus patients was not statistically significant *(P = 0.34)*.

### Risk factors related to strabismus

Table 3 shows the risk factors associated with strabismus. Tilting one’s head when writing (*P* = 0.004) had inverse relationships with the prevalence of strabismus. The odds of strabismus with incorrect writing posture was 1.58 (95% CI:1.16–2.15) times more than correct writing posture, defined as keeping the head in a plane perpendicular to the ground. The effects of age, sex, and ethnicity were not statistically significant (*P*>0.05). In multiple logistic regression for strabismus, writing posture was statistically significant in strabismus subjects (OR1.5; 95% CI, 1.07–2.08), with a higher prevalence of strabismus with incorrect writing posture (tilting one’s head) when writing.

**Table 3:** Risk factors associated with strabismus

Characters	*P*	OR(95%CI)
Age	0.9865	1.00 (0.59–1.69)
Gender	0.8177	0.94 (0.58–1.54)
Ethnic	0.5948	0.56 (0.14–2.32)
Height	0.5436	1.01 (0.97–1.06)
Weight	0.1509	1.04 (0.98–1.11)
BMI	0.1413	1.10 (0.97–1.24)
Heartrate	0.3455	0.99 (0.98–1.01)
Oxygen saturation	0.9967	1.00 (0.92–1.08)
Lamp use	0.2778	0.83 (0.59–1.16)
Writing posture	0.0040	1.58 (1.16–2.15)
Sleep habits	0.9422	1.04 (0.32–3.40)
Pregnancy history	0.6757	1.20 (0.51–2.83)
Fruits intake	0.8290	1.07 (0.58–1.96)
Vegetables intake	0.7230	1.12 (0.61–2.04)

### Refractive status

**Fig. 3:**
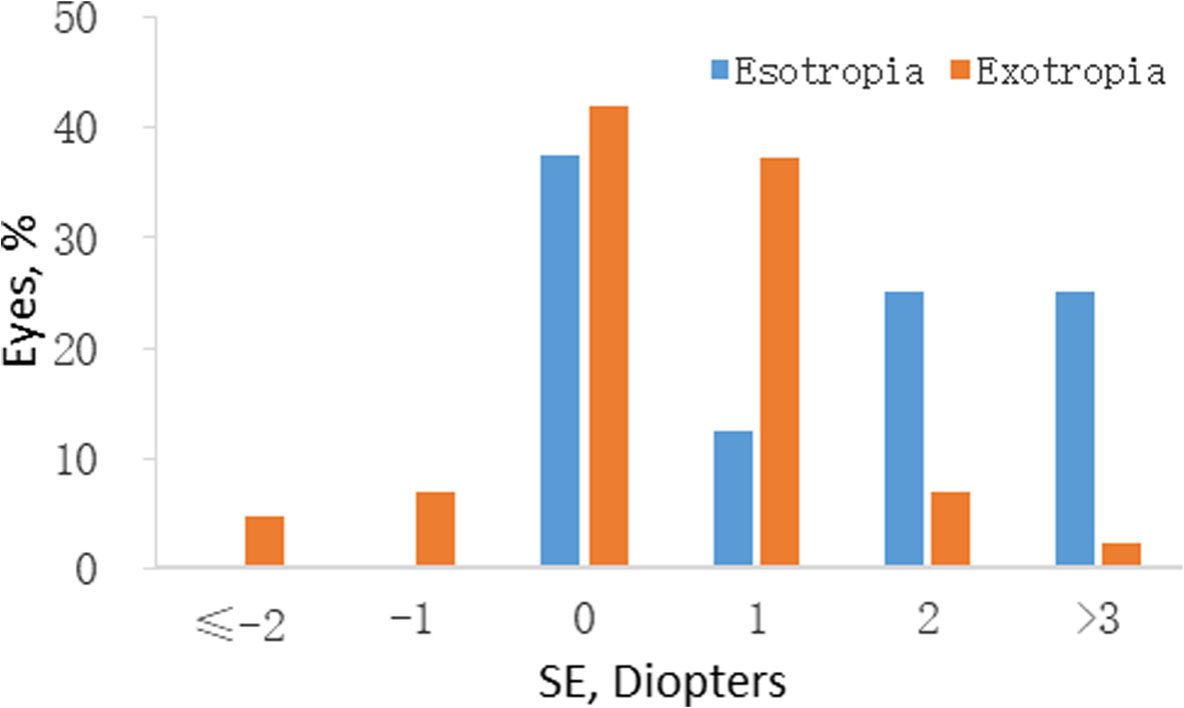
Distribution of the cycloplegic SE of esotropia and exotropia students (right eyes)

Among the 1856 Grade one students examined, 1853 (99.84%) had cycloplegic autorefraction performed on both eyes. The mean cycloplegic SE of the right and left eyes for horizontal strabismus were + 1.24 ± 1.49D and + 1.21 ± 1.33D respectively, with no significant difference (*P* = 0.89). The prevalence rates of emmetropia (between − 0.50D and + 2.00D in both eyes) were 89.15% based on right eye data. The mean cycloplegic SE of esotropia students was + 2.05D compared with + 1.09D in exotropia students; the difference was statistically significant (*P*<0.05). Figure 3 outlines the cycloplegic SE of esotropia and exotropia students.

### Stereopsis

Stereopsis acuity and deviation angle were further evaluated in 49 esotropia and exotropia participants. The mean deviation angle of four participants with impaired stereopsis vision was 35.00 ± 17.80 PD, and for the remaining 45 participants with normal stereopsis vision, was 33.00 ± 11.40PD. Participants who lack stereopsis vision had significantly larger deviation angles than those who have binocular vision *(P < 0.05)*. Participants with stereopsis impairment showed a significant difference between esotropia (3/8) and exotropia (2/43) *(P = 0.026)*, while esotropia was more likely to affect stereopsis.

## Discussion

To the best of our knowledge, the LCES is the first comprehensive school-based cohort study in the high-altitude plateau Lhasa, Tibet, China and provide baseline data of the Tibetan Minority for epidemiology of Grade one students eye disorders. We found the prevalence of strabismus was 3.66% with an exotropia: esotropia ratio was 5.38:1, and participants with esotropia were more likely to affect stereopsis, with most being hyperopic.

The overall prevalence of strabismus in our study was higher than previous reports from China and most of other studies around the world. We summarized the previous prevalence of Chinese pediatric strabismus study in Table 4 [3, 7, 16–23]. The prevalence of strabismus in our study was lower than the 5.65 and 5.8% in different 3–6-year-old children from the Nanjing Pediatric study [7, 18]; Nanjing is a metropolis in eastern China. Compared with the prevalence from other countries, the prevalence of LCES was higher than the 3.3% in Caucasian and 2.1% in African American children in the Baltimore Pediatric Eye Disease Study [24], 2.4% in Hispanic/Latino and 2.5% African-American children in the Multi-Ethnic Pediatric Eye Disease Study [25], 1.3% in Japan [26], 2.4% in Turkey [27], and 0.8% in the Strabismus, Amblyopia and Refractive Error in Singaporean Children (STARS) [28].

**Table 4:** Prevalence of strabismus in previous Chinese studies

Author	Year	Area	Samples	Age/years	Prevalence/%	Exo:Eso
Zhu H	2019	Yunnan	1656	7–8	1.50	10.6:1
			1394	13–14	2.44	
Pan CW	2017	Yunnan	3122	6–8	2.47	
			4305	9–11	3.69	9.4:1
			1836	12–14	4.96	
Pan CW	2016	Nanjing	5831	3–6	5.8	/
Chen X	2016	Nanjing	5884	3–6	5.65	6:1
Fu J	2014	Anyang	3112	7–8	2.73	6:1
			2362	12–13	5.02	51:1
Pi LH	2012	Chongqing	3079	6–15	0.29	1.7:1
Lu P	2008	Qinghai	1084	6–14	2.49	5.7:1
He M	2007	Yangxi	2454	13–17	1.63	3.5:1
He M	2004	Guangzhou	4364	5–15	1.9	4:1
Zhao J	2000	Shunyi	5884	5–15	2.8	/

The higher prevalence of strabismus in LCES may relate to several reasons. Firstly, among the 1856 participants, 94.93% were native Tibetan Minority. The potential influence of ethnic, environmental, lifestyle and genetic susceptibility differences [16] is still unclear. Qian reported that the prevalence of ocular disease differences in Tibet may relate to more exposure to sunlight and easier schooling [29] Whether Tibetan children have a higher prevalence of strabismus requires further investigation. Secondly, different age ranges across the different studies may play an important role [3, 16]. The mean age of our voluntary students was 6.83 ± 0.46 years, and the age range was narrower than other studies, although, older than the two Nanjing pediatric studies [7, 18]. Finally, we recruited four professional strabismus and pediatric ophthalmologists to examine the participants. They have professional experience in checking strabismus, with a likely greater detection rate of less obvious strabismus and mildly impaired visual acuity.

The ratio of exotropia: esotropia ratio in LCES was 5.38:1, which was lower than many studies [3, 7, 16–23]. The reason for this probably relates to the status of refraction; the distribution of the cycloplegic SE of esotropia in LCES tend to be hyperopia, which was consistent with the finding that exotropia was associated with astigmatism, myopia and low to moderate hyperopia, while esotropia was associated with hyperopia in a dose-response manner [16, 30, 31]. Qian also found that Tibetan adolescents had a lower prevalence of refractive errors than the plains (central China) area except for hyperopia, and also suggested that the rate of hyperopia in Tibet is higher [29]. As we show here, in the present LCES study the prevalence of hyperopia was higher at 6.91%. However, the prevalence of hyperopia was 2.21% in ACES. Comparing the significant hyperopic prevalence difference between LCES and ACES, this probably explains the discrepancy in the ratio of exotropia: esotropia between the two studies.

Strabismic amblyopia typically influences the dominance of the fixating eye and chronically reduced responsiveness to input by the non-fixating eye [8]. The mean BCVA of the strabismus and non-strabismus eyes showed students with strabismus had worse BCVA. We also found that participants with stereopsis impairment had bigger deviation angles and esotropia was more likely to affect stereopsis. The VA in strabismic amblyopia can be improved after successful treatment in younger ages and stereopsis can be restored after surgical alignment in many strabismus cases [32, 33] The earlier that clinically significant refractive error and strabismus are detected and treated, the greater the likelihood of preventing or reducing amblyopia.8 However, most children with strabismus and amblyopia in LCES were not diagnosed earlier, let alone treated.

Some studies found higher prevalence of strabismus in older students [3, 16]. Our study did not find that for students in LCES from Grade 1. However, the age span was not as large as others. The effect of some regional factors like oxygen saturation and ethnicity characters were not statistically significant likewise, which is in agreement with the previous study [16]. Dietary (fruits and vegetables) intake did not influence the prevalence of strabismus, either. Tilting one’s head when writing was a risk factor for the prevalence of strabismus. In a forward multiple logistic regression for strabismus, writing posture was statistically significant, reflecting a higher prevalence of strabismus with habits like tilting one’s head when writing. Bao and co-authors reported that the near heterophoria state can affect near vision posture, while head tilt angle and ocular gaze angle had a potential relationship with strabismus [34]. However, it is possible that head tilt may have developed because of strabismus, and can be accepted as a sign of misalignment. Hence, although we consider that tilting one’s head when writing may be a risk factor for the prevalence of strabismus, it requires further research to verify or refute this observation.

There are some limitations to the present study. Some risk factors might be potentially inaccurate due to the self-reported questionnaires from the students’ parents, even though the questionnaires used in the LCES were calibrated for cultural differences. In addition, strabismus history and treatment were not always remembered by their parents clearly, which could lead to a mis-estimate for prevalence of strabismus.

## Conclusion

As the first comprehensive school-based cohort study in Tibet, the LCES had high response rate and good comparability with other studies. Moreover, the LCES study is the first longitudinal study on childhood ocular diseases in plateau regions of Chinese children and will continue follow-up for 5 years and could provide invaluable ophthalmic baseline data for this population and worldwide eye care.

## Supplementary Information

**Additional file 1.**

## Data Availability

The datasets used and/or analysed during the current study available from the corresponding author on reasonable request.

## Abbreviations

LCES: The Lhasa Childhood Eye Study
ACES: The Anyang Childhood Eye Study
BMI, BMI: weight/height^2^ kg/m^2^ Body Mass Index
BCVA: Best-corrected visual acuity
SE: Spherical equivalent
SOP: Superior oblique muscle paralysis
DDP: Double depressor paralysis
DVD: Dissociated vertical deviation
STARS: The Strabismus, Amblyopia and Refractive Error in Singaporean Children
SD: Standard deviation
CI: Confidence interval
PD: Prism diopters
