# Supplementary material for: Prevalence and associated risk factors for childhood strabismus in Lhasa, Tibet, China: a cross-sectional, school-based study
Source: BMC Ophthalmol. 2020 Nov 25;20:463. doi: 10.1186/s12886-020-01732-2 (PMC7687812; doi:10.1186/s12886-020-01732-2)
Supplement: Supplementary file 1 — Additional file 1. [file 12886_2020_1732_MOESM1_ESM.pdf]

No.:______

School:______

Grade:______

Class:______

Name:________

Birth:_______

Gender:_____

Date:________

# The Lhasa Childhood Eye Study Questionnaires

(English Version)

***<u>Completing this questionnaire before your child's get the ophthalmology examination. Please complete it as carefully and accurately as possible.</u>***

**<u>Section1:</u>**

1. Has your child had a vision test in the past year?

2. Do the examination results show that your child's eyesight is abnormal?

3. Have children had regular eye check-ups in the past year?

4. If the child is currently myopic, the age at which the child is diagnosed with myopia is _ years old.

5. Does your child wear glasses at present?

If you wear it, the age at which the child begins to wear glasses is _ years old, and the current cost of glasses is _ yuan.

6. Due to inappropriate or other reasons, the frequency your child's glasses being replaced is?

7. If your child is myopia, do you think he or she should wear glasses?

8. How often do you take your child to visit an ophthalmologist or optometrist?

9. If the doctor advises you to dilate your child's pupil, will you agree?

10. The average cost of each visit to an ophthalmologist or optometrist is?

11. Whether the family members (the students' parents) wear glasses?

13. In the past years, has your child ever received the following myopia treatment?

14. Has your child had eye surgery in the past year?

15. Has a doctor ever told you that your child has a squint?

16. Have you noticed that your child has a strabismus problem?

17. Has an ophthalmologist ever told you that your child has amblyopia?

18. Has your child ever worn glasses for amblyopia?

19. Has your child ever worn a therapeutic blindfold?

**Section2:**

20. When your child is reading or writing, please estimate the distance from the child's face to the book.

21. Does your child have the habit of tilting his head when holding a pen and writing?

23. When your child is watching TV, what is the distance between your seat and the TV?

24. When your child uses the computer, what is the distance from the computer?

25. What is the distance between your child's seat and the screen when playing video games?

26. When your child is studying, what kind of light bulb do you use?

27. How long does your child usually stop to have a rest after reading or doing close work?

28. How long has your child been absent from school for various reasons in the past year? (please make a rough estimate).

29. From Monday to Friday, in addition to school, the average daily study time for children is _ hours?

30. On weekends, the average daily study time for your child is _ hours?

31. In general, how long does your child take a break?

32. Does your children attend outdoor sports in their spare time?

33. Does your children attend indoor sports in their spare time?

34. Do you think eye exercises can prevent myopia and protect eyesight?

35. Do you think eye exercises can reduce visual fatigue?

**Section3:**

36. Did your child born full term?

37. Did your child breathe oxygen after birth?

38. Is the city where your child has been living before?

**Section4:**

39. Does your child turn on the light when he goes to bed at night?

40. If your child is in the habit of turning on the lights when he or she goes to bed at night, which light bulb is used?

41. Your family lives on the _ floor, and the living area is _ square meters.

42. What time can the sun shine in the house in the morning?

43. How far can you see from the child's bedroom window?

**Section5:**

45. How many vegetables does your child usually eat every day? Vegetables include all fresh, dried, frozen and canned vegetables.

46. How much fruit does your child usually eat every day? Includes all fresh, dried, frozen and canned fruit.

**Thank you very much for cooperating with us to complete this questionnaire, we will keep your answers confidential, and we look forward to your child's coming to attend the ophthalmology examination!**
